# The Mechanical Analysis of the Biofilm Streamer Nucleation and Geometry Characterization in Microfluidic Channels

**DOI:** 10.1155/2016/7819403

**Published:** 2016-05-30

**Authors:** Xiaoling Wang, Mudong Hao, Xin Du, Guoqing Wang, Jun-ichi Matsushita

**Affiliations:** ^1^School of Mechanical Engineering, University of Science and Technology Beijing, Beijing 100083, China; ^2^School of Engineering and Applied Sciences, Harvard University, Cambridge, MA 02138, USA; ^3^Laboratory of Nonlinear Mechanics, Institute of Mechanics, Chinese Academy of Sciences, Beijing 100190, China; ^4^Department of Materials Science, Tokai University, Hiratsuka 259-1292, Japan

## Abstract

Bacteria can form biofilm streamers in microfluidic channels with various geometries. Experiments show that the streamer geometry, such as its shape or thickness, depends on the fluid velocity and the geometry and curvature of the microfluidic channel. In the paper, a mechanical analysis of the flow field is made in different channels, which shows that the secondary flow in the channel is the reason for streamer nucleation and that the shear stress distribution decides the streamer geometry including shape and thickness. Through a finite elements simulation, we obtain the secondary flow forming positions in both static and rotating channels: positions that are the location of nucleation of the streamer. Thick or wide biofilm streamers occur at the points of minimum shear stress in static channels. Furthermore, in rotating channels, spiral-like streamers form, due to the helical shape of the minimum shear stress distribution. The findings may allow the prevention of biofilm formation and also the removal of bacteria adhered onto certain surfaces in channels with small cross sections. The analysis also indicates how one can obtain desirable biofilm streamers by control of the channel geometry and the loading conditions.

## 1. Introduction

Biofilms are natural structures formed by microbial communities encapsulated inside a matrix of self-secreted extracellular polymeric substances (EPS), growing most commonly on a solid surface [1, 2]. Biofilms strongly interact with their environments and can form highly complex morphologies and adapt to a wide variety of environmental conditions [3]. The abundant presence of biofilms has important implications: for example, they are used in waste-water treatment plants for the removal of pollutants [4]. In waste-water pipes, one observes the formation of biofilms in the form of thread-like structures called “streamers.” Better understanding of the streamer formation and its critical influences in channels would be helpful in waste-water decontamination.

**Figure 1:**
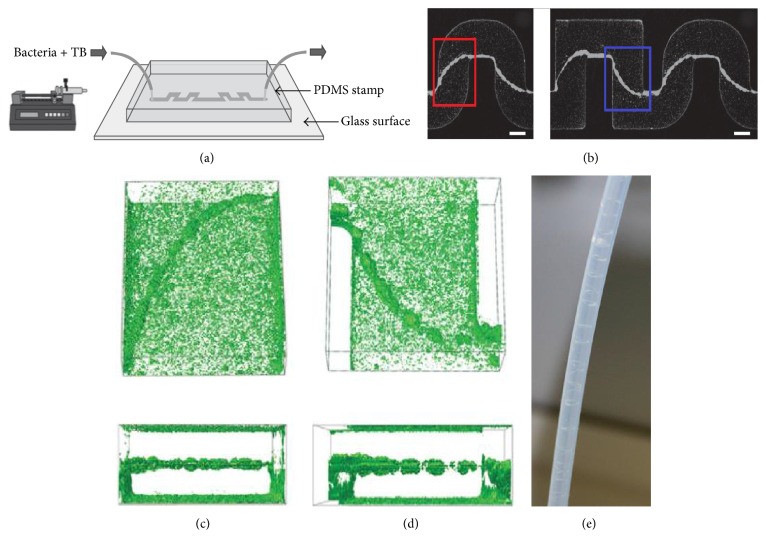
Experimental evidence for bacterial thread-like biofilms (streamers) in curved microchannels. (a) Schematic of the microfluidic device in which bacterial solutions, containing cells and nutrients, flow through the channel for several hours. The width of the channel is 200 *μ*m and the typical height is about 100 *μ*m. (b) Bacterial streamers developed in the channels after 12 h at constant flow rate (corresponding to an average speed of 0.75 *μ*L/min) for two different experiments. The flow direction is from left to right. Bacteria are fluorescently labeled, and images of the middle plane of the channel are taken with a confocal microscope. Scale bars, 100 *μ*m. (c) Three-dimensional reconstruction from* z*-scan confocal images and cross-sectional views are shown (red box in (b) depicts observed location). (d) Three-dimensional reconstruction from* z*-scan confocal images and cross-sectional views are shown (blue box in (b) depicts observed location). (e) The helicoidal bacterial threads (streamers) formed in the inner wall of circular channels. Images of (a)–(d) are cited from Rusconi et al. [12]. Image of (e) is cited from Rodríguez Espeso 2013. TB is tryptone broth. The microfluidic channels were prepared from polydimethylsiloxane (PDMS, Sylgard 184 silicone elastomer kit, Dow Corning) following conventional soft-lithography techniques.

The formation of the biofilm streamers in fluid flow is affected by various factors such as surface roughness, materials, temperature, and pH [5–7]. With the development of microfluidics technology for biological applications, past researchers have noticed that the geometry of the microfluidic channel could influence streamer formation and thickness [8–10]. Mehdi Salek et al. found that the biofilm formed in the square channel is thicker than that formed in a rectangular channel, as shown in Figures 1(a) and 1(b) [11]. Rusconi et al. observed biofilm streamer formation in the middle of channels with different corner shapes; the streamer initially formed at the corners and then stayed connected only to the lateral walls while the rest of the streamer structure lay suspended in the flow, as shown in Figures 1(c) and 1(d) [12]. Rodríguez Espeso observed the formation of long helicoidal bacterial threads (streamers) wrapped around the inner walls of circular channels, when the channel rotates while the bacterial solution flows through it, as shown in Figure 1(e) [13].

Numerical simulation showed that one possible reason for biofilm streamer formation around corners in microfluidic channels is secondary flow, which is a relatively minor flow superimposed on the primary flow. Rusconi et al. showed that the wall shear stress is one of the major factors of biofilm formation [12]. Quantitative analysis of the dependence of streamer nucleation position and streamer structure on the fluid and stress fields in various microfluidic channels, however, is lacking.

The experimental observations cited above clearly show that the external environment has important effects on biofilm formation. In particular, shear force plays a role in biofilm streamer formation in pipes and sewer lines [14]. But the quantitative relationship between biofilm streamer structure and shear force distribution is lacking, pointing to the need for theoretical work and numerical simulation for a more complete understanding of streamer formation. In the work, the Fluent CFD software is used to calculate the shear force distribution in various fluid channels. The results show that the secondary flow in the channel is the reason for biofilm streamer nucleation and that the shear stress distribution decides biofilm streamer geometry including its shape and thickness.

## 2. Theory: Fluid Mechanics

We obtain numerical solutions of the flow field in these geometries via finite-element simulations of the incompressible form of the Navier-Stokes equations and mass conservation (continuity) equations by using Fluent CFD software. The velocity field $\vec{U}$ and pressure *p* can be obtained from the complete set of governing equations in terms of fluid density *ρ* and viscosity *μ*:

(1) ρU→·∇U→=−∇p+μ∇2U→,∇·U→=0.

Steady state was assumed in all cases, so there is no time-dependent partial derivative in (1). No-slip and no-flux were assumed along the walls of the channel. The flow rates were set to ensure that once the channels converged, the average flow velocity in the main channel, *U*_*m*_, resulted in the specified Reynolds number, expressed in terms of channel cross-sectional area *A*, average volumetric flow rate *Q*, the fluid density *ρ*, fluid viscosity *μ*, and the channel hydraulic diameter *D*_*h*_:

(2) Um=QA,ReD=ρUmDhμ,Dh=4AΓ,Γ=2b+H.

At the low flow rates that were investigated in this study, the flow remains laminar. That being the case, the shear stress was approximated as

(3) $$\begin{array}{c} \tau =\mu \frac{dU_{m}}{dy}. \end{array}$$

## 3. Numerical Simulation and Results

The biofilm streamer formation is considered in both straight channels and curved channels and also considered both static and rotating channels to explore the dependence of biofilm formation and streamer structure on shear force distribution in various channels.

### 3.1. Biofilm in the Static Straight Channel

**Figure 2:**
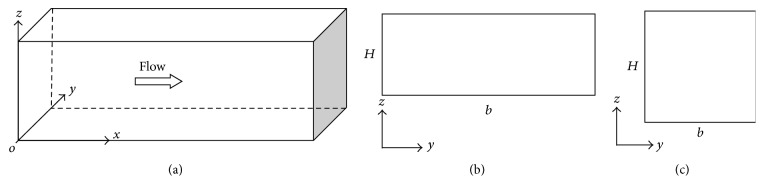
(a) Schematics of the microfluidic channel; (b) the microfluidic channel with the rectangular cross section; (c) the microfluidic channel with the square cross section.

We set up straight channels with two different cross sections: square and rectangular, as shown in Figure 2. The main flow parameters used in this study are summarized in Table 1. For comparison we use the same flow condition at constant value of *U*_*m*_ (mm/s).

**Figure 3:**
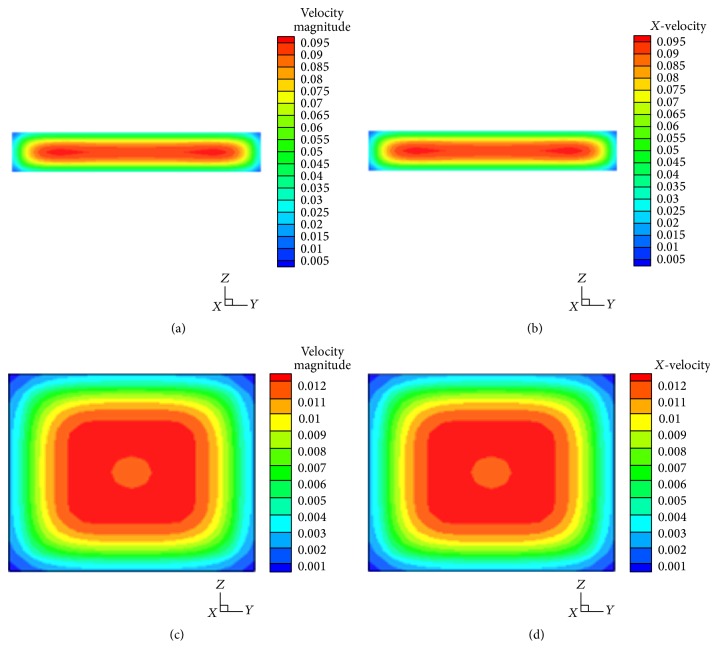
Contours of velocity (m/s) in cross section of channels. (a) Contours of velocity magnitude (m/s) in a cross section of rectangular channel; (b) contours of* X*-velocity (m/s) in a cross section of rectangular channel; (c) contours of velocity magnitude (m/s) in a cross section of square channel; (d) contours of* X*-velocity (m/s) in a cross section of square tube channel.

The contour velocity distributions of straight channels with rectangular and square cross sections are shown in Figure 3. The lateral and vertical velocity components are almost zero for both square and rectangular cross section channels.

**Figure 4:**
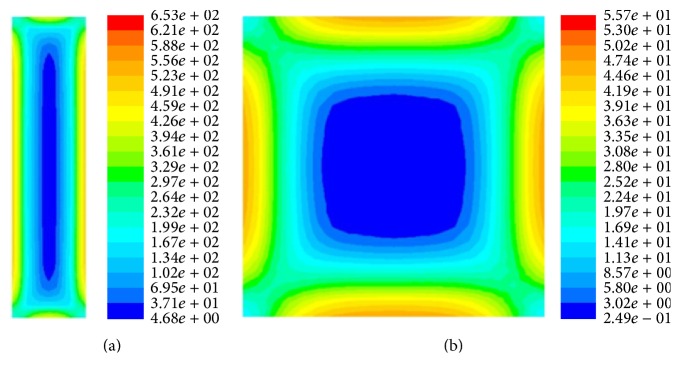
Contours of the strain rate (1/s) in cross section of channels. (a) Contours of the strain rate (1/s) in a cross section of rectangular tube channel; (b) contours of the strain rate (1/s) in a cross section of square tube channel.

In order to determine the relationship between shear stress and biofilm thickness, we calculate the shear strain rate which is proportional to shear stress, as shown in (3). The shear strain rate for the rectangular cross section is larger than that for the square cross section. In both cases, the higher shear rate region is located near the center of the walls, while lower shear rate region is located by the corners, as shown in Figure 4.

**Figure 5:**
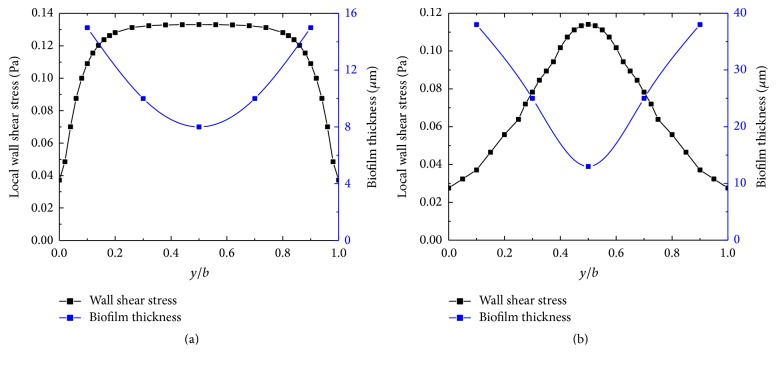
The distribution of the shear stress along the base wall for both the rectangular and square channels. (a) The distribution of the shear stress along the base wall for the rectangular channel. The blue line indicates the experimental data of biofilm thickness in rectangular channel; (b) the distribution of the shear stress along the base wall for the square channel. The blue line indicates the experimental data of biofilm thickness in square channel.

The shear stress distribution along the base wall for both the rectangular and square channels is indicated by the black line in Figure 5. The corresponding experimental data of biofilm thickness is indicated by the blue line in Figure 5 [11]. The result shows a negative correlation between local shear stress and biofilm thickness for both cross sections. Thicker biofilm is located near the corner where the shear stress is low, while thinner biofilm is located near the center of the wall which has the higher shear stress.

**Figure 6:**
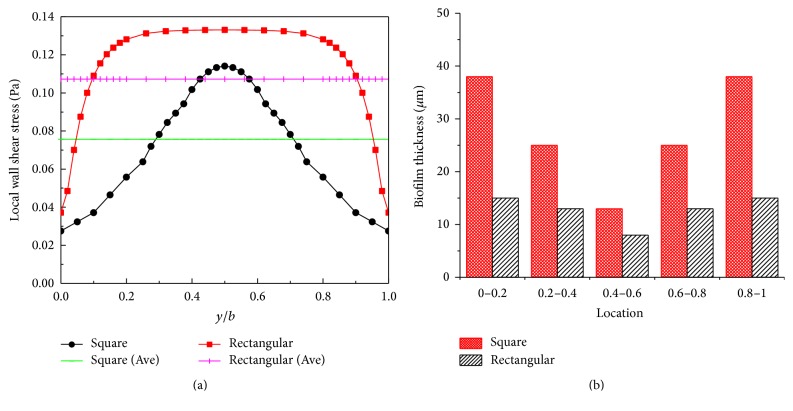
The distribution of the wall shear stress along the base wall for both the rectangular and square channels and the laboratory measured the thickness of the biofilm. (a) The distribution of the wall shear stress along the base wall for the rectangular channel. The pink line indicates average value of wall shear stress in rectangular channel; the cyan line indicates average value of wall shear stress in square channel. (b) The experimental data of biofilm thickness in rectangular and square channel.

The average shear stress for the two different crosses is indicated by pink and green lines in Figure 6(a): the average shear stress is higher for the rectangular channel, which results in a thicker biofilm in the square channel, as shown in Figure 6(b) [11].

### 3.2. Biofilm in the Rotating Straight Channel

**Figure 7:**
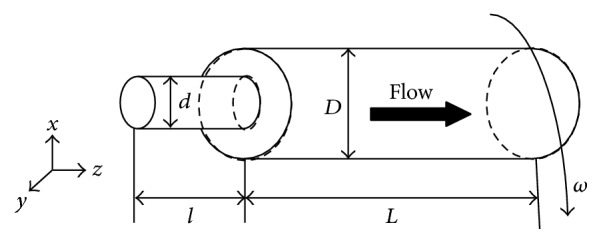
Schematics of the cylindrical channel. The flow direction is from left to right.

Rotating straight channels of different diameters with the bacterial solution flowing through are considered, as shown in Figure 7. Although roller pumps setup in Rodríguez Espeso's experiment [13] were based on a traditional peristaltic pump, the working mechanism of roller pumps here is slightly different from the peristaltic pump. Roller pumps push the fluid forward by straining the channel containing the fluid with the rollers; the rotation of the rollers achieves angular flow velocity. So the roller pumps can achieve both the forward flow velocity and angular flow velocity.

In our simulation, we use rotating channels to get angular flow velocity replacing the tube rotation driven by the connected roller. We set a steady forward fluid flow velocity replacing the one by straining the channel containing the fluid with the rollers and finally get the similar working condition in experiment. As our focus is on the shear stress which decides the structure of biofilm streamers, we appropriately simplify the simulation without changing the streamer formation mechanism in the experiment. In our simulation, channels are rotating when the bacteria solution steady flow through, instead of the pulsatile flow in the experiment, which was produced by the roller pump. The parameters used in this case are summarized in Table 2 from Rodríguez Espeso's experiment [13].

**Figure 8:**
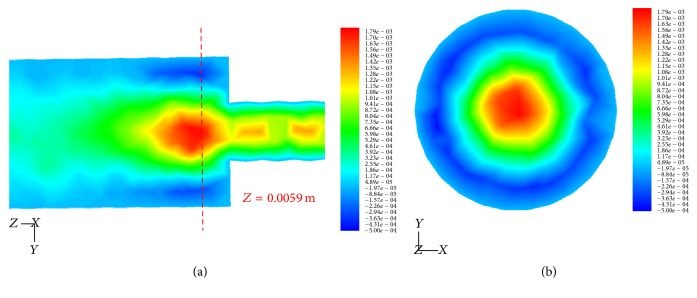
Contours of* Z*-velocity (mm/s) in different cross-section of the cylindrical channels. (a)* Z*-velocity field (mm/s) in* Y-Z* plane at *X* = 0. (b)* Z*-velocity field (mm/s) in* X-Y* plane at *Z* = 5.9 mm.

The experimental observations also show that the biofilm nucleated in the joint connecting channels with different diameters, wherein the significant secondary flow from the numerical simulations is located as shown in Figure 8(a). The biofilm formed helicoidal threads near the inner wall of the rotating channel; the* Z*-velocity (mm/s) is lowest because of secondary flow as shown in Figure 8(b).

**Figure 9:**
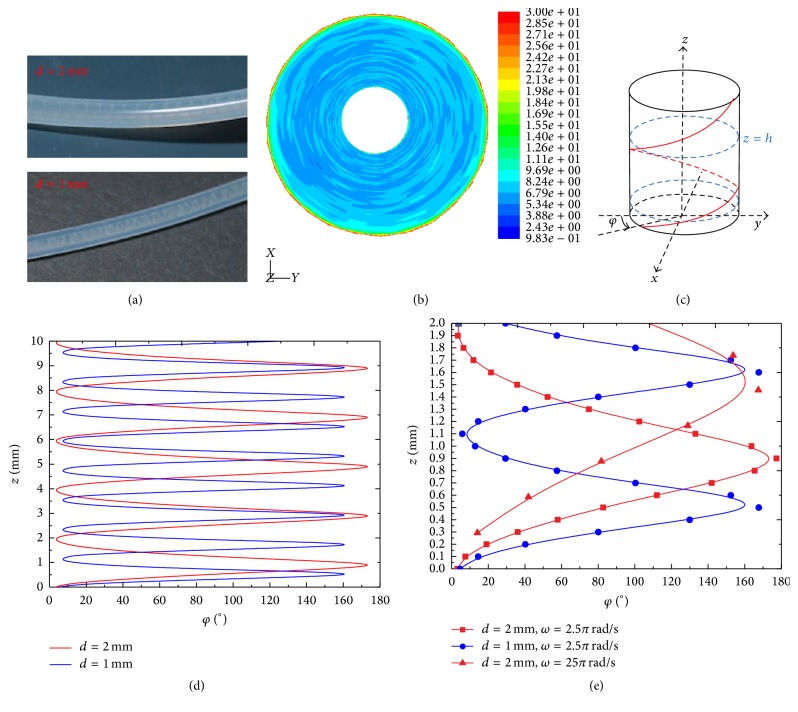
(a) Helicoidal biofilm streamers with different helicoidal size steps in cylindrical channels in diameters of 1 mm and 2 mm. (b) The spiral shape of the contour of minimum stain rate near the inner wall of the cylindrical channel (upward view). (c) Schematic of the line joining points of the minimal shear stress on different circular cross sections of the channel. (d) The spiral-like biofilm streamer in the cylindrical channel projected on a* Y-Z* flat surface. (e) An enlarged region of the helicoidal biofilm streamers, the blue line with circular points and the red line with red square points indicate biofilm streamers, formed in channels with diameters of 1 mm and 2 mm under the same volumetric flow rate and angular rotation velocity; the red line with circular points and the red line with red triangular points indicate helicoidal biofilm streamers formed in the same diameter channels but under different angular rotation velocities.

Rodríguez Espeso observed the formation of long helicoidal bacterial threads (streamers) wrapped around the inner wall of circular channels, when the channels rotate while the bacterial solution flows through [13]. For the same volumetric flow rate and the rotation angular velocity the helicoidal streamer size step depends on the diameter of channels, as shown in Figure 9(a) [13].  Figure 9(b) shows the distribution of the strain rate around the inner wall of the channels where the helicoidal biofilm streamers occur. The experimental observation is schematically shown in Figure 9(c); for the arbitrarily chosen cross section *z* = *h*, the angle *φ* is from the negative* Y* direction to the line between the minimum shear strain point and the cross section center. The red spiral line in Figure 9(c) indicates the line connecting the minimal shear strain points on each cross section. The line connecting the minimal shear strain points on each cross section (the arbitrary chosen 10 mm along* Z* direction) projected on a* Y-Z* flat surface is shown in Figure 9(d); the red and blue lines represent the channel diameters of 2 mm and 1 mm, respectively. The helix size step of the red line is longer than that of the blue line, which agrees with the experimental observation, as shown in Figure 9(a). The enlargement figure shows that one helicoidal biofilm streamer size step forms in the 2 mm diameter channel, but under the same volumetric flow rate and the rotation angular velocity we find nearly twice the size in the 1 mm diameter channel, as shown in the blue line with circular points and the red line with red square points in Figure 9(e). We further consider biofilm streamer in the same channels but under different rotation angular velocity; the helicoidal size step decreases with increasing the angular rotation velocity, as shown in the red line with circular points and the red line with red triangle points of Figure 9(e).

### 3.3. Biofilm in Curved Channels

In this section, we consider a flow in a channel of constant rectangular cross section, which exhibits a 90° turn that is characterized by a sharp corner along the flow direction. Furthermore, we also explain the relationship between shear stress and biofilm streamer formation around corners with different angles (210°, 240°, and 270°).

**Figure 10:**
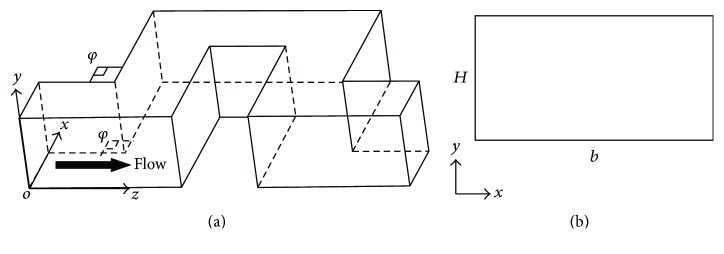
(a) Schematics of the 3D microfluidic channel; (b) the microfluidic channel with the rectangular cross section.

We established a curved shape channel model, as shown in Figure 10. The main flow parameters used in this study are summarized in Table 3.

Since the Reynolds number is rather small (in the range 0.02–0.1), the flow in the microchannels is everywhere laminar. We used commercial finite-element software (Fluent) to perform three-dimensional numerical simulations of the flow in curved channels with the same geometry and physical parameters as used in the experiments.

**Figure 11:**
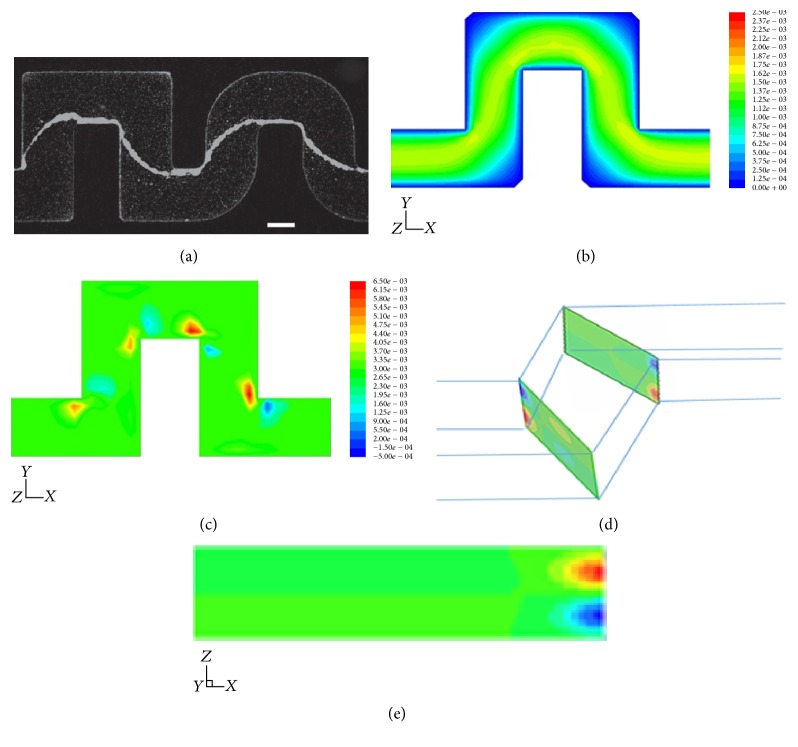
(a) Bacterial streamers developed in the channels after 12 h at constant flow rate (corresponding to an average speed of 0.75 *μ*L/min) for two different experiments. The flow direction is from left to right. Bacteria are fluorescently labeled, and images of the middle plane of the channel are taken with a confocal microscope. Scale bars, 100 *μ*m. (b) Numerical results of the modulus of the velocity field in a plane at 1/4 of the channel height from the upper surface. Coordinate system (*x*, *y*, *z*) and color scale are shown. (c) Flow components perpendicular to the primary flow (represented here with streamlines) in the same plane as (b). Red and blue colors indicate here velocity components orthogonal to the plane of the channel, directing, respectively, upwards (positive* y*) and downwards (negative* y*). (d) Perspective view of the channel showing pairs of counterrotating vertices in cross-sectional planes right before and after the turns. (e) A cross section of the channel, right after a turn.

The experimental phenomena are displayed in Figure 11(a). The main features of the results are shown in Figures 11(b)–11(e). In Figure 11(b), contour plots of the modulus of the velocity field |**u**| are shown in the middle plane of the channel (*z* = 1/2). The primary flow matches the flow pattern predicted in a two-dimensional planar geometry: a contour plot of the velocity field and the associated streamlines, in a plane at a quarter of the channel height from the upper surface, are displayed, respectively, in Figures 11(c)-11(d).  Figure 11(d) shows contour plots of the secondary flow, obtained from 3D numerical simulations in the proximity of the corners both rounded and sharp. This secondary flow consists of two symmetrical counterrotating vortices of length scale comparable to half the channel height, as shown in Figure 11(e).

In addition, we also set up different corner angle (210°, 240°, and 270°) 3D microfluidic channels model according the experiment. The parameters of the model are consistent with the elbow-bend pipe model parameters in addition to the corner angle being different.

**Figure 12:**
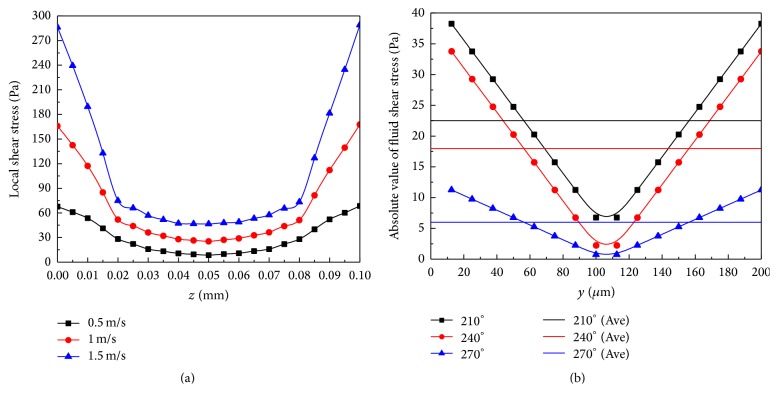
(a) The local shear stress of the channel with a 210° corner turn for different flow rates, that is, 0.5 m/s, 1 m/s, 1.5 m/s. (b) Absolute value of fluid shear stress in the microfluidic channel with different corner angles (210°, 240°, and 270°).

Rusconi et al. obtained the average width by measuring the area covered by the streamers and found that the greater the flow velocity, the smaller the width of the streamer in the microfluidic channel with certain corner angles [12]. From our simulations, we get local shear stress for different flow velocities. The local shear stress increases with the fluid velocity; then the high shear stress decreases the streamer width in the microfluidic channel with certain corner angles, as shown in Figure 12(a). Besides, the experiments also revealed that the greater the corner angle, the larger the width of the streamer in the microfluidic channel. We find that the increase of corner angle at the same flow velocity reduces the local shear stress, resulting in a larger width of the streamer in the channel, as shown in Figure 12(b).

## 4. Discussion and Conclusion

In our work, we make the mechanical analysis on the flow and shear stress fields in various channels to find the dependence of biofilm nucleation position on the fluid velocity distribution; what is more, we also find that the biofilm streamer thickness formed in static channel on flow velocities and different corner angle. Our results show that the shear stress can significantly influence biofilm thickness and shape by changing the hydrodynamics of the local environment surrounding the biofilm streamer in these channels. These simulation results explain the experimental observations very well. The conclusions are as follows.

*(1) Biofilm Streamer in the Static Straight Channel.* The biofilm streamer is formed in the bottom wall in straight channels for both rectangular and square cross sections; what is more the thick biofilm streamer distributes near the inner corner of the channel where the shear stress is lower; the thin biofilm preferring to distribute instead near the center bottom wall where shear stress is higher. The actual shear stress will be higher than in the simulation because the biofilm streamer reduces the cross-sectional area, with a subsequent rise in flow velocity and shear stress.

*(2) Biofilm Streamer in the Rotating Straight Channel*. The biofilm streamer nucleates near the joint connecting two cylindrical channels with different diameters because of the secondary flow forming at the joint. Interestingly, the approximate helicoidal distribution of the shear stress near the inner wall can induce the helicoidal bacterial streamer formation in the rotating cylindrical channel. Although there are several possible locations which correspond to minimum shear stress for different cross sections of the rotating cylindrical channels, only the minimum distance between two minimum shear stress positions from the adjacent cross sections can guarantee minimum energy consumption. Integrally connecting all the above minimum distances along cylindrical channel forms the spiral-like biofilm streamer.

From the careful observation of the helicoidal bacterial streamer in experiment, which is shown in Figure 1(e) [13], the size step and the thickness of streamer are not uniform, which is because the minimum shear stress distribution induced by the pulsatile flow is heterogeneous; the pulsatile flow has different flow rates in each cycle along the channel, while in our simulation we get the uniform the size step and the thickness of streamer by using steady flow. However the streamer formation mechanism is the same in experiment and our simulation; the streamer structure is mainly decided by the minimum shear stress distribution on the channel.

*(3) Biofilm Streamer in the Static Curved Channels.* The biofilm streamer nucleates in the position where the secondary flow forms; the biofilm streamer width along *X* direction is decided by shear stress along *X* direction; this width increases with increasing the curvature of the channels.

In a word, the shear stress distribution in channels is the key factor to the biofilm streamer structure. The fundamental study on shear stress shows a way to prevent biofilm formation and further removal of adhered bacteria on the certain surface in narrow channels. Further, we can obtain the desirable biofilm streamer by controlling the channel geometry and the loading conditions.

## Figures and Tables

**Table 1:** Flow conditions for rectangular and square cross section channel.

	*L* (mm)	*b* (mm)	*H* (mm)	*U*_*m*_ (mm/s)	*D*_*h*_ (mm)
Rectangular	100	5	1	0.01	1.67
Square	100	2	2	0.01	2

**Table 2:** Flow conditions for the cylindrical channel.

*L* (mm)	*l* (mm)	*D* (mm)	*d* (mm)	*U*_*m*_ (mm/s)	*Q* (mL/min)	Re_*D*_	*ω* (rad/s)
40	5	2	0.8	0.795	0.15	1.591	2.5*π*
40	5	1	0.8	3.183	0.15	3.183	2.5*π*

**Table 3:** Flow conditions for 90° curved shape channel model.

	*L* (*μ*m)	*b* (*μ*m)	*H* (*μ*m)	*U*_*m*_ (mm/s)	*D*_*h*_ (*μ*m)
Value	600	200	100	0.75	133.3
